# Differential contribution of Ca^2+^ sources to day and night BK current activation in the circadian clock

**DOI:** 10.1085/jgp.201711945

**Published:** 2018-02-05

**Authors:** Joshua P. Whitt, Beth A. McNally, Andrea L. Meredith

**Affiliations:** Department of Physiology, University of Maryland School of Medicine, Baltimore, MD

## Abstract

Large conductance K^+^ (BK) channels are expressed widely in neurons, where their activation is regulated by membrane depolarization and intracellular Ca^2+^ (Ca^2+^_i_). To enable this regulation, BK channels functionally couple to both voltage-gated Ca^2+^ channels (VGCCs) and channels mediating Ca^2+^ release from intracellular stores. However, the relationship between BK channels and their specific Ca^2+^ source for particular patterns of excitability is not well understood. In neurons within the suprachiasmatic nucleus (SCN)—the brain’s circadian clock—BK current, VGCC current, and Ca^2+^_i_ are diurnally regulated, but paradoxically, BK current is greatest at night when VGCC current and Ca^2+^_i_ are reduced. Here, to determine whether diurnal regulation of Ca^2+^ is relevant for BK channel activation, we combine pharmacology with day and night patch-clamp recordings in acute slices of SCN. We find that activation of BK current depends primarily on three types of channels but that the relative contribution changes between day and night. BK current can be abrogated with nimodipine during the day but not at night, establishing that L-type Ca^2+^ channels (LTCCs) are the primary daytime Ca^2+^ source for BK activation. In contrast, dantrolene causes a significant decrease in BK current at night, suggesting that nighttime BK activation is driven by ryanodine receptor (RyR)–mediated Ca^2+^_i_ release. The N- and P/Q-type Ca^2+^ channel blocker ω-conotoxin MVIIC causes a smaller reduction of BK current that does not differ between day and night. Finally, inhibition of LTCCs, but not RyRs, eliminates BK inactivation, but the BK β2 subunit was not required for activation of BK current by LTCCs. These data reveal a dynamic coupling strategy between BK channels and their Ca^2+^ sources in the SCN, contributing to diurnal regulation of SCN excitability.

## Abstract (precis)

Large conductance K^+^ (BK) channels require intracellular Ca^2+^ provided by Ca^2+^ channels to open at physiological membrane potentials. Whitt et al. identify the Ca^2+^ channel subtypes that activate BK current in the suprachiasmatic nucleus to enable time of day to be encoded by the circadian clock.

## Introduction

Large conductance K^+^ (BK) channels (K_Ca_1.1), encoded by the *KCNMA1* gene, are critical regulators of membrane potential in a variety of excitable cells. BK channels are activated via membrane depolarization and increases in local intracellular Ca^2+^ (Ca^2+^_i_; Fakler and Adelman, 2008; Latorre et al., 2017). Like voltage, calcium sensitivity is intrinsic to the BK channel pore-forming α subunit (Wu et al., 2010; Yuan et al., 2010). Although BK channels can open in response to either stimulus, in the context of most excitable cells, both stimuli are required to gate channel opening (Fakler and Adelman, 2008; Latorre et al., 2017). Micromolar concentrations of Ca^2+^_i_ are required to shift the voltage-dependence of activation into the physiological range of membrane potentials (≤50 mV; Prakriya et al., 1996; Pérez et al., 2001; ZhuGe et al., 2002). These relatively high concentrations are achieved through tight functional coupling to voltage-gated Ca^2+^ channels (VGCCs) or channels mediating Ca^2+^ release from intracellular stores in local nanodomains (Nelson et al., 1995; Prakriya et al., 1996; Marrion and Tavalin, 1998; Pérez et al., 2001; ZhuGe et al., 2002; Berkefeld et al., 2006). However even with high local Ca^2+^_i_, in smooth muscle cells the β1 subunit is necessary to enhance the Ca^2+^-dependent gating required for BK channel activation (Brenner et al., 2000b). Similarly, the neuronally expressed β2 and β4 subunits shift the conductance–voltage relationship to more hyperpolarized potentials (Wallner et al., 1999; Xia et al., 1999; Brenner et al., 2000a; Meera et al., 2000).

BK-Ca^2+^ channel coupling is multifarious, but incompletely understood. Differing across tissues, BK channels have been shown to be activated by L-type (Roberts et al., 1990; Wisgirda and Dryer, 1994; Prakriya and Lingle, 1999; Sun et al., 2003; Berkefeld et al., 2006; Berkefeld and Fakler, 2008; Marcantoni et al., 2010; Bellono et al., 2017; Vivas et al., 2017), N-type (Robitaille et al., 1993; Wisgirda and Dryer, 1994; Yazejian et al., 1997; Marrion and Tavalin, 1998; Sun et al., 2003; Loane et al., 2007), P/Q-type (Prakriya and Lingle, 1999; Edgerton and Reinhart, 2003; Womack et al., 2004; Goldberg and Wilson, 2005; Berkefeld and Fakler, 2008; Indriati et al., 2013; Lee et al., 2014; Irie and Trussell, 2017), and T-type (Smith et al., 2002; Rehak et al., 2013) VGCCs, as well as intracellular store–mediated Ca^2+^ release through IP3 receptors and ryanodine (Ryan) receptors (RyRs; Neely and Lingle, 1992; Herrington et al., 1995; Nelson et al., 1995; Bolton and Imaizumi, 1996; Prakriya et al., 1996; Chavis et al., 1998; Jaggar et al., 1998; Merriam et al., 1999; Akita and Kuba, 2000; ZhuGe et al., 2000; Wang et al., 2016; Irie and Trussell, 2017). Other Ca^2+^-permeable channels have also been implicated in BK activation (Hu et al., 2001; Isaacson and Murphy, 2001; Wu et al., 2013). Physical associations have been demonstrated for BK and L-type (Cav1.2 and Cav1.3), P/Q-type (Cav2.1), and N-type (Cav2.2) channels (Grunnet and Kaufmann, 2004; Berkefeld et al., 2006; Loane et al., 2007; Berkefeld and Fakler, 2008; Vivas et al., 2017). BK channels are also spatially localized with IP3 and RyR intracellular store release channels (Marcotti et al., 2004; Beurg et al., 2005; Weaver et al., 2007; Kaufmann et al., 2009; Irie and Trussell, 2017). Although the subcellular localization of BK-Ca^2+^ channel complexes may determine the influence of BK channel opening on excitability, such as their contribution to action potential properties versus presynaptic neurotransmitter release, the relative contribution of different Ca^2+^ sources to BK activation within the same subcellular compartment is not well understood. Many cells express BK channels alongside several Ca^2+^ channel types, but the consequences of using multiple Ca^2+^ sources in BK’s regulation of specific aspects of excitability is just beginning to be explored (Prakriya and Lingle, 1999; Berkefeld and Fakler, 2008; Wang et al., 2016).

In the suprachiasmatic nucleus (SCN) of the hypothalamus, the brain’s internal “clock,” BK currents are present alongside all the major VGCC and intracellular Ca^2+^ channel types. Interestingly, a paradoxical relationship exists between BK current and Ca^2+^_i_. Cytosolic Ca^2+^_i_ undergoes oscillations, peaking in the middle of the day (Colwell, 2000; Ikeda et al., 2003; Enoki et al., 2012, 2017a,b; Hong et al., 2012; Brancaccio et al., 2013). In contrast, BK current magnitude is largest at night (Meredith et al., 2006; Pitts et al., 2006; Whitt et al., 2016), indicating the specific Ca^2+^ sources regulating BK activation require additional investigation. L-, N-, P-, Q-, R-, and T-type VGCCs and intracellular IP3 receptors and RyRs are all present in SCN neurons (Hamada et al., 1999; Pennartz et al., 2002; Cloues and Sather, 2003; Jackson et al., 2004; Kim et al., 2005; Nahm et al., 2005; Aguilar-Roblero et al., 2007;). L-type channels contribute to the larger daytime VGCC currents observed in SCN neurons, and firing decreases when L-type Ca^2+^ channels (LTCCs) are inhibited during the day (Pennartz et al., 2002; Cloues and Sather, 2003; Jackson et al., 2004), suggesting these channels play a role in the circadian modulation of firing rate. Genetic alterations of both L-type and N-type VGCC function alter circadian rhythm (Masutani et al., 1995; Beuckmann et al., 2003; van Oosterhout et al., 2008; Nakagawasai et al., 2010; Huang et al., 2012; Schmutz et al., 2014). Cytosolic Ca^2+^_i_, potentially mediated by RyR release of Ca^2+^ from intracellular stores (Ikeda et al., 2003; Ikeda and Ikeda, 2014; Aguilar-Roblero et al., 2016), is also higher during the day. However, the contribution of RyR-mediated Ca^2+^ release to the circadian modulation of firing rate has produced heterogeneous results (Aguilar-Roblero et al., 2007, 2016). Nevertheless, RyRs have also been implicated in the regulation of circadian behavioral rhythms (Ding et al., 1998; Mercado et al., 2009). Despite the circadian modulation of Ca^2+^ in SCN neurons, the convergent regulation of multiple sources of Ca^2+^ influx contributed by plasma membrane and intracellular channels, and their contribution to the daily oscillation in action potential firing, is not clear.

In this study, we investigated which Ca^2+^ sources are responsible for BK current activation in SCN neurons and whether there is evidence that circadian regulation of BK-Ca^2+^ coupling contributes to the day-versus-night difference in BK current properties and action potential firing (Meredith et al., 2006; Whitt et al., 2016). The effects of pharmacological inhibition and activation of VGCC and intracellular Ca^2+^ channels were assessed by whole-cell recordings of BK currents and action potentials during the day and the night in acute SCN slices. We identified a time-of-day trend to BK activation by different Ca^2+^ sources, with corresponding changes in firing frequency that suggest Ca^2+^ may regulate SCN excitability in part through modulation of BK channel activity.

## Materials and methods

### Mice

WT and β2 knockout (KO) mice (provided by C. Lingle; Martinez-Espinosa et al., 2014) on a C57BL/6J background were used at 3–6 wk old from males and females housed (from birth) on a standard 12:12-h light–dark cycle (day time points) or a reverse 12:12-h light–dark cycle (night). All procedures involving mice were conducted in accordance with the University of Maryland School of Medicine Animal Care and Use Guidelines and approved by the Institutional Animal Care and Use Committee.

### Electrophysiology

Acute SCN slices were prepared as described (Whitt et al., 2016), at Zeitgeber time (ZT) 0:00–2:00 (day) or ZT11:00–12:00 (night) and recovered 1–3 h in oxygenated artificial cerebrospinal fluid (in mM: 125 NaCl, 1.2 MgSO_4_, 26 NaHCO_3_, 1.25 Na_2_HPO_4_, 3.5 KCl, 2.5 CaCl_2_, and 10 glucose) at 25°C. SCN neurons were recorded in whole-cell configuration as described (Whitt et al., 2016) from neurons in the center of the SCN at the peak (ZT4:00–8:00) and nadir (ZT17:00–21:00) of the circadian rhythm in spontaneous firing. Data were acquired at 50 or 100 kHz with a MultiClamp 700B amplifier and filtered at 10 kHz. Electrodes (4–7 MΩ) were filled with internal solution (in mM): 123 K-methanesulfonate, 9 NaCl, 0.9 EGTA, 9 HEPES, 14 Tris-phosphocreatine, 2 Mg-ATP, 0.3 Tris-GTP, and 2 Na_2_-ATP, pH 7.3. Membrane properties were elicited from a 20-mV step from a holding potential of −90 mV. R_a_ was <25 MΩ with less than ±5% change (on average ∼15 MΩ). R_s_ was compensated at 80%.

In voltage-clamp mode, total voltage-activated K^+^ currents were recorded in 1 µM tetrodotoxin (TTX; 1069; Tocris) ± focally perfused Ca^2+^ channel modulators: 10 µM nimodipine (Nim; P-450; Alomone), 10 µM dantrolene (Dan; D9175; Sigma), 5 µM thapsigargin (TG; T-650; Alomone), 3 µM ω-conotoxin MVIIC (MVIIC; C-150; Alomone), 5 µM Bay K 8644 (BayK; B133; Sigma), or 100 nM Ryan (559276; Sigma). Stocks were prepared at 1,000× in DMSO (Nim, Dan, TG, BayK, paxilline [Pax]) or water (MVIIC, Ryan, TTX). Currents were elicited from a holding potential of −90 mV, stepping from −110 to 90 mV for 150 ms in 20-mV increments. BK currents were isolated by current subtraction after focal application of the BK antagonist 10 µM Pax (Alomone). Three currents were averaged per cell and normalized to cell capacitance, which did not differ between day and night or between WT and β2 KO (Whitt et al., 2016). Voltage values were adjusted for the liquid junction potential (10 mV). Inactivating (BK_i_) and sustained (BK_s_) currents were categorized by using the current ratio (peak to 30 ms) from 90-mV step (BK_i_ < 0.07, BK_s_ > 0.07), corresponding to τ_inact_ values <110 ms. V_1/2_ values were determined from Boltzmann fits of the I/I_max_ relationships (Origin 8.5; OriginLab).

For Ca^2+^ current recordings in whole-cell voltage-clamp mode, the internal solution was (in mM) 115 cesium gluconate, 10 tetraethylammonium chloride, 10 HEPES buffer, 0.5 EGTA, 2 MgCl_2_, 20 sodium phosphocreatine, 2 Na_2_ATP, and 0.3 Na_3_ GTP, pH 7.3, and bath (mM) 68 NaCl, 3.5 KCl, 1 NaH_2_PO_4_, 26.2 NaHCO_3_, 1.3 MgSO_4_, 2.5 CaCl_2_, 10 D(+)-glucose, 60 tetraethylammonium chloride, and 3 CsCl (pH 7.4) and 1 µM TTX. R_s_ was compensated at 60%. Inward Ca^2+^ currents were elicited by using the same voltage protocol as for BK currents (in 10-mV increments) at baseline and after 10 µM Nim or 3 µM MVIIC. Three currents were averaged per cell and normalized to cell capacitance.

In current-clamp mode, action potentials were recorded by using the same intracellular and bath solutions as for K^+^ currents, and the data were acquired in 10-s sweeps. Frequency was calculated as the mean of each sweep. Input resistance and resting membrane potential were recorded in 1 µM TTX and calculated as previously described (Whitt et al., 2016).

### Statistics

Group means are reported ± SEM. Reported numbers in figure legends are the number of neurons recorded, with 1–6 neurons per animal. Data for each condition was derived from a minimum of two animals. Statistical significance was determined at P < 0.05 by using Origin (OriginLab) or SPSS v19 (IBM) for unpaired Student’s *t* test for pairwise comparisons, one-way ANOVA for comparisons between control and multiple drug conditions (p-values reported in the text are the Bonferroni post-hoc tests only for comparisons where the main effect was P < 0.05), and Fisher’s exact test categorical data (i.e., number of BK_i_ versus BK_s_ neurons).

### Online supplemental material

Fig. S1 shows data for passive membrane properties of day and night SCN neurons with Ca^2+^ channel inhibitors. Fig. S2 contains a comparison of the relative inhibition of BK current from WT and β2 KO SCN neurons during the day and night in Nim and Dan. Fig. S3 is a schematic model depicting a hypothesis for functional coupling of BK and Ca^2+^ channels between day and night SCN neurons. 

## Results

### Voltage-activated Ca^2+^ currents are larger during the day in SCN neurons

Multiple membrane parameters are diurnally modulated in the mammalian SCN (Kuhlman and McMahon, 2006; Colwell, 2011). In the whole-cell recording configuration from acute brain slices, we verified the day (recorded at 4–8 h after lights on) versus night (17–21 h after lights on) difference in the spontaneous firing rate (day: 2.07 ± 0.10 Hz, *n* = 65; night: 0.51 ± 0.05 Hz, *n* = 65; P < 0.05, *t* test), resting membrane potential (V_m_ day: −49.1 ± 0.9 mV, *n* = 20; night: −55 ± 1 mV, *n* = 18; P < 0.05, *t* test), and input resistance (R_i_ day: 1.55 ± 0.09 GΩ, *n* = 20; night: 0.77 ± 0.07, *n* = 18; P < 0.05, *t* test), when firing was abolished by the application of 1 µM TTX. Inhibition of BK channel activity has been shown to regulate each of these parameters, with more significant effects at night compared with during the day (Fig. S1; Meredith et al., 2006; Pitts et al., 2006; Kent and Meredith, 2008; Whitt et al., 2016).

**Figure 1.**
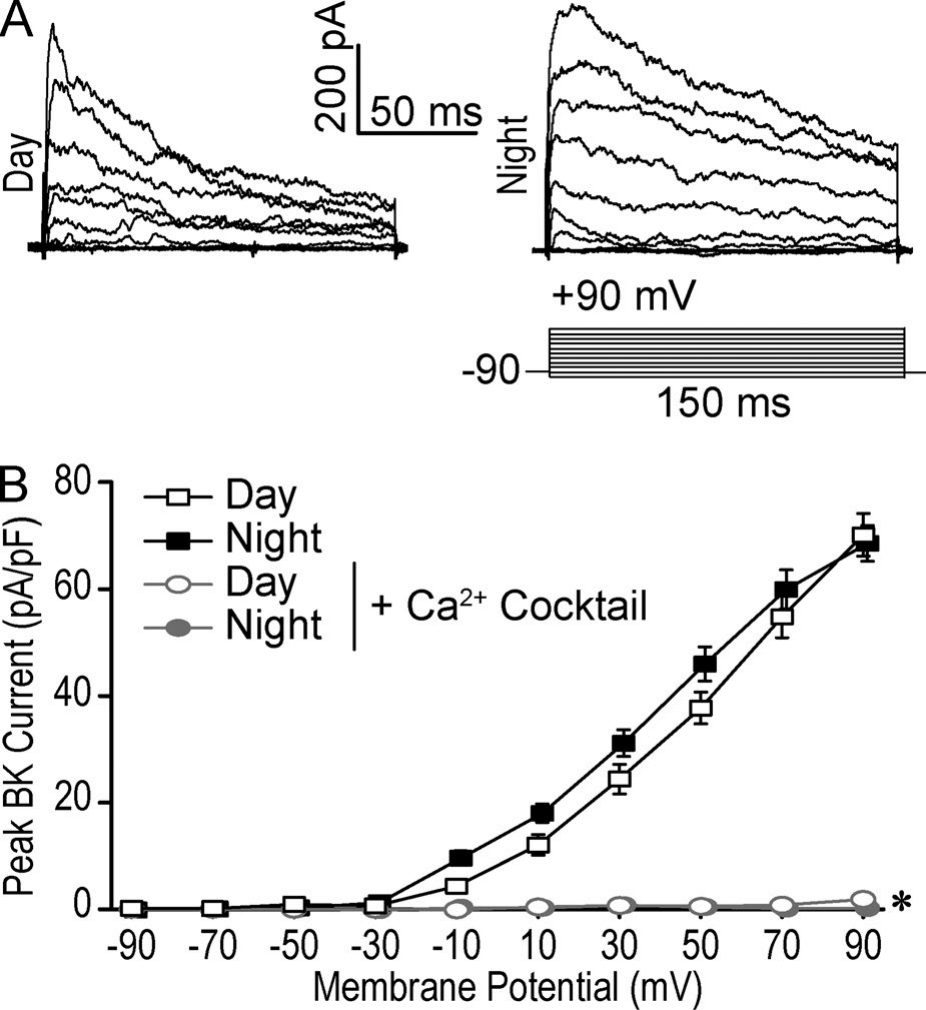
**BK current activation requires Ca^2+^ influx in SCN neurons.** Macroscopic voltage-activated currents were recorded in whole-cell voltage-clamp mode from WT SCN neurons during the day (4–8 h) or night (17–21 h). The pipette (internal) solution contained 0.9 mM EGTA, which allows stable recordings in the presence of Ca^2+^ influx from endogenous channels (Jackson et al., 2004; Fakler and Adelman, 2008; Montgomery et al., 2013; Whitt et al., 2016). From a holding potential of −90 mV, currents were elicited from 150-ms voltage steps in 20-mV increments. BK currents were isolated by subtracting currents elicited in 10 µM Pax from baseline. **(A)** Representative daytime and nighttime BK currents. The daytime current is BK_i_ (τ_inact_ < 110 ms), and the nighttime current is noninactivating BK_s_ (τ_inact_ > 110 ms). **(B)** BK current versus voltage relationship in day and night SCN neurons and under Ca^2+^ channel inhibition. BK current levels were quantified from peak values. The Ca^2+^ cocktail contained 10 µM Nim, 10 µM Dan, and 3 µM MVIIC. Data are mean ± SE. *, BK current value at 90 mV: One-way ANOVA with Bonferroni post hoc (day and night versus cocktail: P = 10^−15^ and 10^−15^, respectively). Day, *n* = 24 neurons; night, *n* = 25; day (Ca^2+^ cocktail), *n* = 10; night (Ca^2+^ cocktail), *n* = 6.

Concomitant with the nighttime-biased role in membrane properties, BK currents are increased at night, with the foremost difference observed in steady-state current levels (Pitts et al., 2006; Montgomery et al., 2013; Whitt et al., 2016). Although the increase in nighttime BK current could be due to an increase in the Ca^2+^ that activates BK channels, several lines of evidence argue against this. First, it was previously shown that inactivation of BK channels during the day accounts for the day-versus-night difference in steady-state BK current levels, and relief from inactivation accounts for the primary increase in BK current levels at night (Whitt et al., 2016). Moreover, quantifying the peak of the evoked BK current reveals a blunted day-versus-night difference (Fig. 1, A and B), suggesting similar initial BK activation at both times of the cycle. Lastly, both VGCC current (a local Ca^2+^ source) and cytosolic Ca^2+^_i_ levels have been reported to be higher during the day than during the night (Díaz-Muñoz et al., 1999; Colwell, 2000; Ikeda et al., 2003; Enoki et al., 2017a). This incongruous relationship between BK activation and Ca^2+^_i_ levels motivated a direct examination between BK channels and their Ca^2+^ source across the circadian cycle.

**Figure 2.**
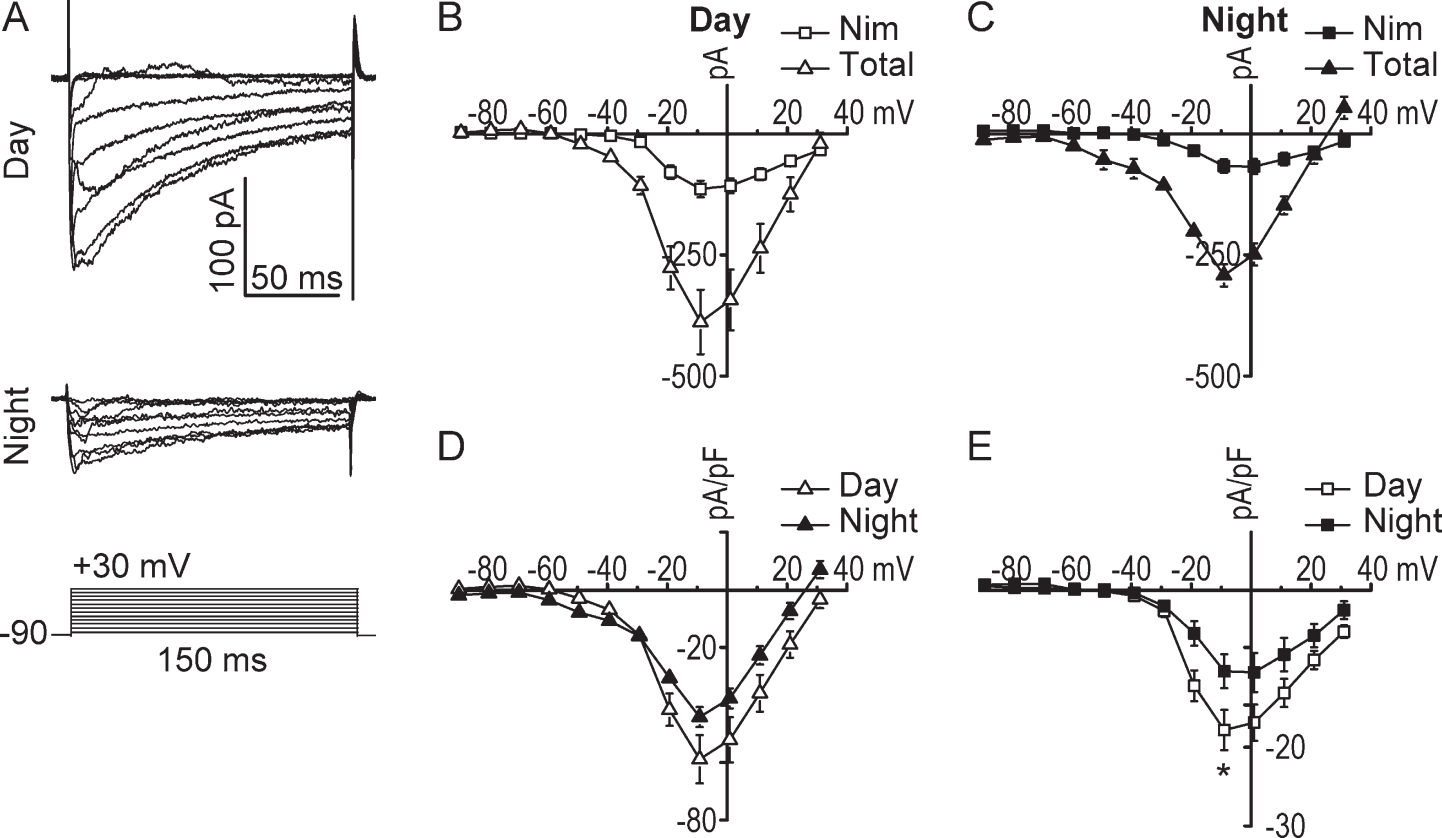
**VGCCs in SCN neurons. (A)** Representative daytime and nighttime Ca^2+^ currents from WT neurons, elicited with the same voltage protocol (in 10-mV increments) used to record BK currents in Fig. 1. **(B and C)** Peak Ca^2+^ current versus voltage relationships before application of 10 µM Nim (total currrent) and the Nim-sensitive current. **(D)** Day-versus-night relationship for the total Ca^2+^ current. **(E)** Day-versus-night relationship for the L-type (Nim-sensitive) Ca^2+^ current. *, One-way ANOVA with Bonferroni post hoc (P = 0.04). Day, *n* = 13 neurons; night, *n* = 9. All data are mean ± SE.

First, because the Ca^2+^_i_ concentrations are not defined in the whole-cell recording configuration, we determined the magnitude of the VGCC current under the same ionic conditions and voltage protocol used to record BK currents in this study (Fig. 2). Macroscopic Ca^2+^ currents were recorded from SCNneurons prepared from daytime and nighttime slices. Application of 1 µM TTX to block Na^+^ current and 60 mM TEA to block voltage-activated K^+^ channels revealed large inward Ca^2+^ currents during the day and night. Similar to previous studies (Pennartz et al., 2002), we found the total daytime Ca^2+^ current was larger than the nighttime current (Fig. 2 D; current at −10 mV, day: −59 ± 8 pA/pF, *n* = 13; night: −44 ± 4 pA/pF, *n* = 9; P > 0.05, *t* test), although the difference was not statistically significant under these conditions.

Because the LTCC current was previously shown to be diurnally modulated in SCN (Pennartz et al., 2002), we applied 10 µM Nim to specifically isolate this current under the conditions in this study. During the day, the LTCC comprised 30% of the total VGCC current, whereas at night, it was 23% (Fig. 2, B and C). The Nim-sensitive LTCC current was larger during the day (current at −10 mV, day: −17 ± 3 pA/pF, *n* = 13; night: −10 ± 2 pA/pF, *n* = 9; P < 0.05, *t* test). In contrast, the N- and P/Q-type Ca^2+^ current, isolated with MVIIC, was not different between day and night (current at −10 mV, day: −10 ± 1.7 pA/pF, *n* = 7; night: −9 ± 1.9 pA/pF, *n* = 7; P > 0.05, *t* test). These results show the activation of VGCC current under conditions used to record BK current is, at least in part, paradoxically inversely phased. Because VGCC and LTCC currents decrease at night without a concomitant decrease in BK current, it suggests that BK activation may be influenced by other mechanisms (Shelley et al., 2013; Whitt et al., 2016), and potentially other Ca^2+^ sources, than only VGCCs.

### BK current activation depends on distinct Ca^2+^ sources during the day and night

We broadly addressed the Ca^2+^ dependence of BK channel activation by recording BK currents in the presence of a cocktail of inhibitors that block both VGCCs (Nim and MVIIC) and intracellular RyRs (Dan), local Ca^2+^ sources that are known to be coupled to BK channel activation in other cell types (Robitaille et al., 1993; Wisgirda and Dryer, 1994; Nelson et al., 1995; Prakriya et al., 1996; Chavis et al., 1998; Marrion and Tavalin, 1998; Merriam et al., 1999; Prakriya and Lingle, 1999; Akita and Kuba, 2000; ZhuGe et al., 2000; Edgerton and Reinhart, 2003; Womack et al., 2004; Goldberg and Wilson, 2005; Berkefeld et al., 2006; Loane et al., 2007; Berkefeld and Fakler, 2008; Marcantoni et al., 2010; Indriati et al., 2013; Lee et al., 2014; Wang et al., 2016; Bellono et al., 2017; Vivas et al., 2017). In SCN neurons, inhibition of each of these Ca^2+^ channel types had previously been shown to modestly alter firing rates, suggesting their function could make some contribution to the circadian patterning of excitability (Pennartz et al., 2002; Cloues and Sather, 2003; Jackson et al., 2004; Aguilar-Roblero et al., 2016). Both during the day and at night, BK currents were essentially completely abolished with the Ca^2+^ channel inhibitor cocktail (Fig. 1 B), demonstrating that one or some combination of L-type, N/P/Q-type, and RyR channels are required for effectively all the BK current activation in SCN neurons at both time points in the circadian cycle.

**Figure 3.**
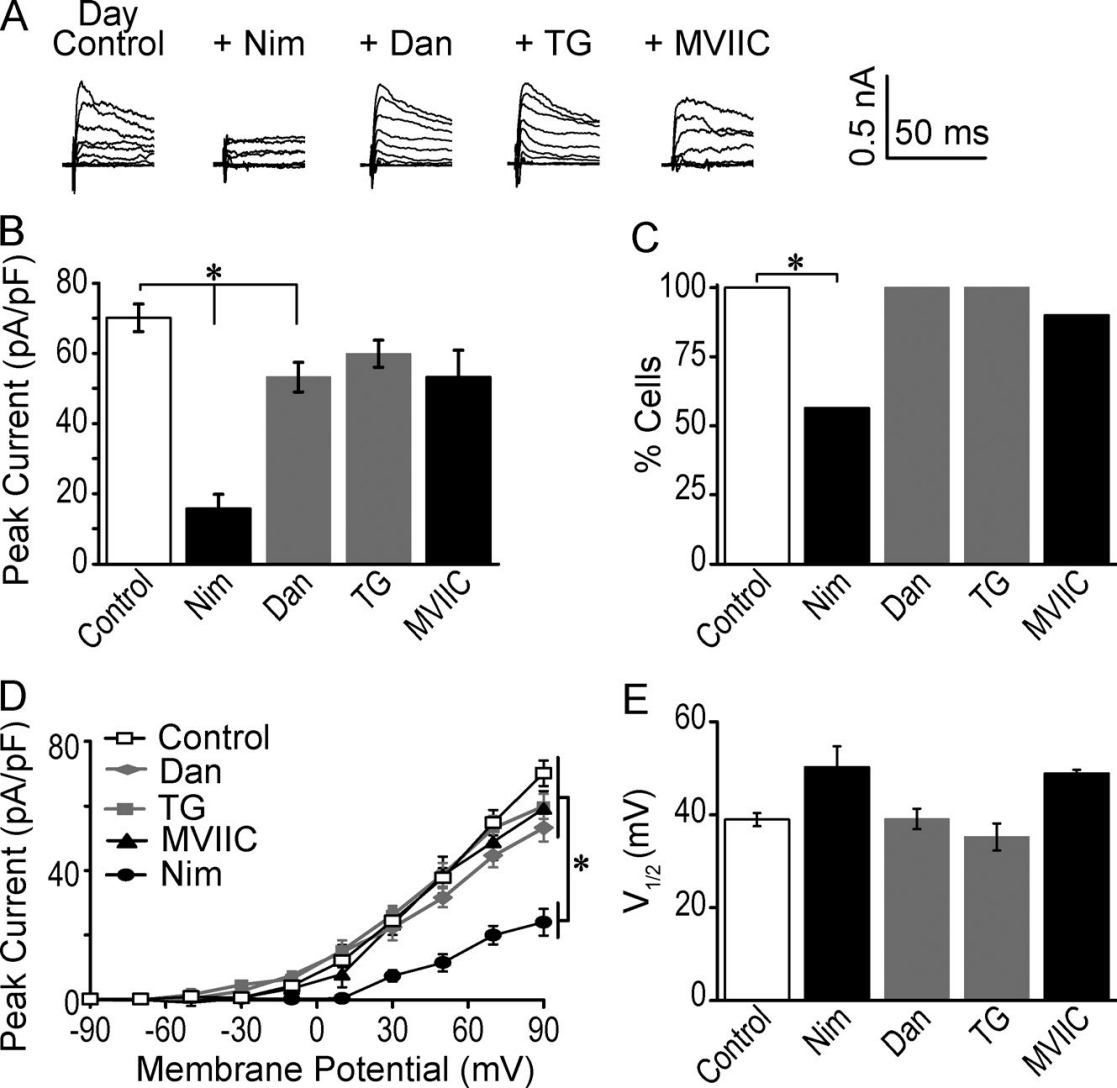
**Effect of inhibition of Ca^2+^ channels on BK currents during the day. (A)** Representative daytime peak BK currents recorded from WT SCN neurons in control conditions or with 10 µM Nim, 10 µM Dan, 5 µM TG, or 3 µM MVIIC. Voltage protocol was applied as in Fig. 1. Traces truncated at 50 ms. **(B)** Peak daytime BK current at 90 mV was reduced in Nim, Dan, and MVIIC compared with control. *, One-way ANOVA with Bonferroni post hoc (P = 10^−13^, 0.04, and 0.04, respectively). At 30 mV, only Nim significantly reduced the BK current (P = 10^−6^). **(C)** The percentage of neurons with a BK current was decreased with Nim. *, P = 10^−6^, Fisher’s exact test. (B and C) Control, *n* = 24 neurons; Nim, *n* = 14; Dan, *n* = 17; TG, *n* = 10; and MVIIC, *n* = 10. **(D)** Peak current-versus-voltage relationship for only those cells exhibiting a BK current in the presence of each inhibitor. *, One-way ANOVA with Bonferroni post hoc (control vs. Nim at 90 mV: P = 10^−8^). At 30 mV, only Nim significantly reduced the BK current (P = 0.006). **(E)** Voltage of half-maximal activation values (V_1/2_) from fits of I/I_max_ relationship of only those cells exhibiting a BK current. No significant differences were obtained in any conditions versus control (one-way ANOVA, P = 0.08). (D and E) Control, *n* = 19 neurons; Nim, *n* = 7; Dan, *n* = 17; TG, *n* = 10; and MVIIC, *n* = 4. All data are mean ± SE.

**Figure 4.**
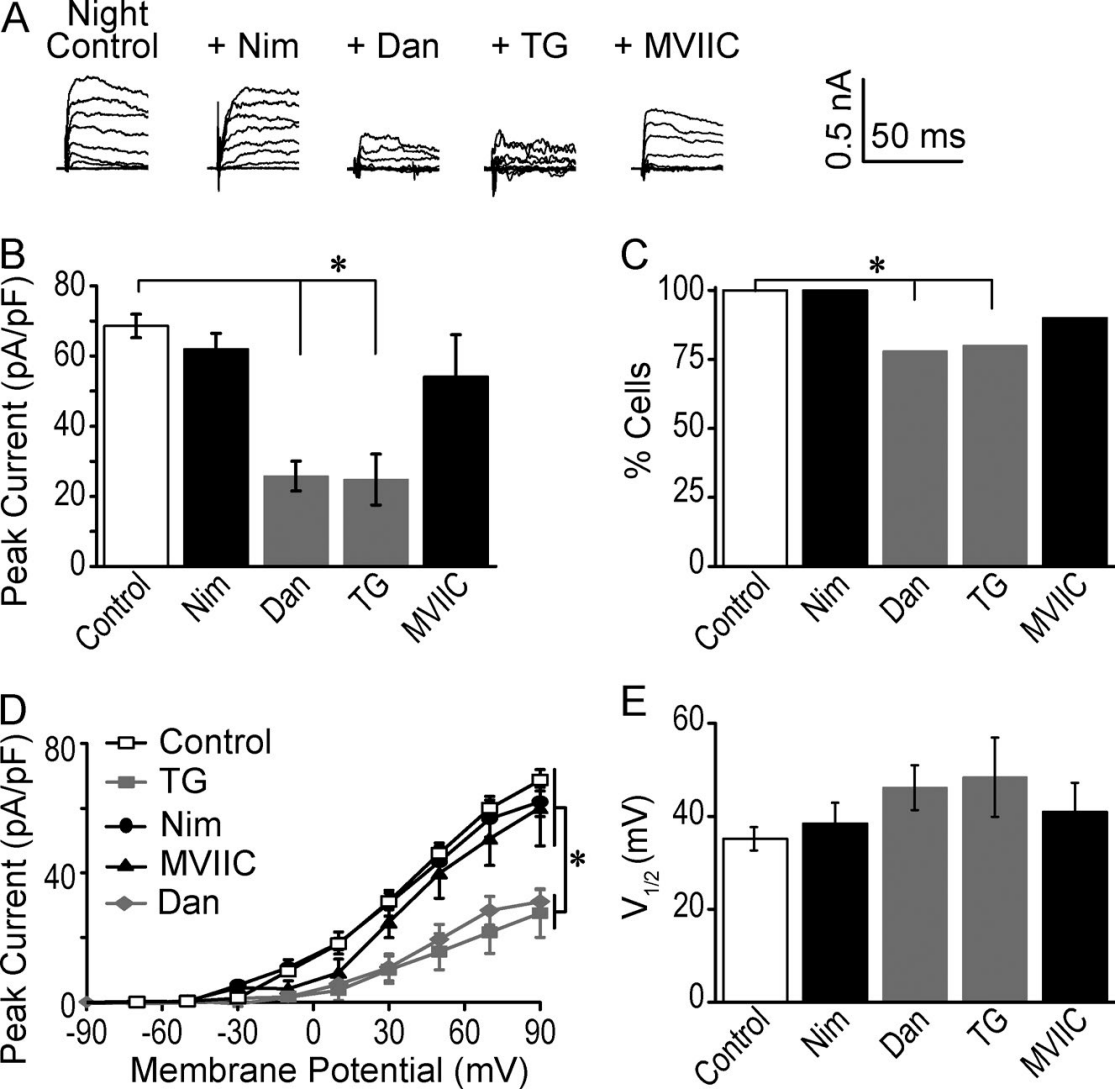
**Effect of inhibition of Ca^2+^ channels on BK currents at night. (A)** Representative nighttime BK currents in control, Nim, Dan, TG, and MVIIC as in Fig. 3. **(B)** Peak BK current at 90 mV was reduced with Dan and TG compared with control. *, One-way ANOVA with Bonferroni post hoc (control vs. Dan at 90 mV: P = 10^−4^; TG: P = 10^−4^). At 30 mV, Dan and TG both significantly reduced the BK current (P = 10^−5^ and 10^−4^, respectively), but Nim did not. **(C)** The percentage of neurons with a BK current was decreased with Dan and TG versus control. *, Fisher’s exact test, Dan: P = 0.03; TG: P = 0.02. There was no difference with the other inhibitors versus control. (B and C) Control, *n* = 25 neurons; Nim, *n* = 17; Dan, *n* = 18; TG, *n* = 10; and MVIIC, *n* = 9. **(D)** Peak current-versus-voltage relationship for only those cells exhibiting a BK current in the presence of each inhibitor. *, One-way ANOVA with Bonferroni post hoc (control vs. Dan at 90 mV: P = 10^−4^; TG: P = 10^−3^). At 30 mV, Dan and TG both significantly reduced the BK current (P = 10^−5^ and 10^−4^, respectively), but Nim did not. **(E)** V_1/2_ values. No significant differences were obtained in any conditions versus control (one-way ANOVA, P = 0.22). Control, *n* = 25 neurons; Nim, *n* = 17; Dan, *n* = 13; TG, *n* = 7; and MVIIC, *n* = 7. All data are mean ± SE.

The role of each Ca^2+^ channel subtype in BK channel activation was next addressed by applying inhibitors individually (Figs. 3 A and 4 A). Peak, rather than steady-state, BK current levels were assessed in this study to eliminate the influence of inactivation. Because of the potential for both voltage-dependent and nonvoltage-dependent Ca^2+^ sources to contribute to BK activation within a single neuron, current levels were assessed at the maximally activating step for BK current (90 mV). During the day, BK currents recorded in Nim were reduced by 77% (Fig. 3 B). This reduction was due to both a decrease in the number of SCN neurons possessing a detectable BK current, from 100% to 56% (Fig. 3 C), as well a decrease in the size of the BK current in the remaining cells (Fig. 3 D). In neurons that still had a BK current in the presence of Nim, the current-voltage relationship was shifted toward more depolarized potentials by 11 mV (Fig. 3 E), although this difference was not significant and probably reflects the partial reliance on other Ca^2+^ channels in these cells. Collectively these results suggest that in almost half of SCN neurons during the day, BK channels rely primarily on LTCCs for their Ca^2+^, whereas the remaining cells contain BK channels activated by both L-type and other Ca^2+^ sources.

At night, Nim had little effect on BK current (Fig. 4, A and B). Furthermore, no neurons exclusively required L-type current for BK current activation at night (Fig. 4 C). Instead, inhibition of Ca^2+^ release from intracellular stores had the largest effect on BK current magnitude. Dan, inhibiting RyR-mediated Ca^2+^ release from intracellular stores (Aguilar-Roblero et al., 2007), and TG, inhibiting SERCA-mediated store refilling, both produced a significant reduction in BK current (64%, Fig. 4 B). This reduction was also due to some neurons exhibiting complete loss of the BK current in Dan (Fig. 4 C), as well as a decreased magnitude in the cells that still had a BK current (Fig. 4 D). In the first group, compared with the number of daytime neurons where BK currents relied solely on LTCCs (∼50%, Fig. 3 C) at night, fewer neurons had BK currents that relied exclusively on intracellular Ca^2+^ stores for activation (∼20%; Fig. 4 C). In the second group, the neurons that did have a BK current in the presence of Dan or TG, the magnitude was about half of the control nighttime level (Fig. 4 D). These BK currents recorded in the presence of Dan or TG also had a positive, but nonsignificant, trend in the voltage-dependence of activation (Fig. 4 E).

Also, similar to BK activation by LTCCs, BK activation by RyRs was different between day and night. However, in contrast to nighttime (Fig. 4 B), Dan and TG applied during the day reduced BK current levels to a much lesser extent (22%, Fig. 3 B). Neither drug decreased the number of neurons with a detectable BK current during the day (Fig. 3 C). From these results, we conclude that during the day, there is a reduced role for RyR-mediated intracellular Ca^2+^ release in BK activation.

Interestingly, inhibition of the N/P/Q-subtypes with MVIIC revealed a similar degree of BK current reduction at both times of day, 21% and 25%, respectively (Figs. 3 B and 4 B). The number of neurons with BK currents that exclusively relied on N/P/Q-type Ca^2+^ channels was not different at either time of day (Figs. 3 C and 4 C). The voltage-dependence of activation was also not significantly different in MVIIC compared with control (Figs. 3 E and 4 E). For the most part, this result suggests that N/P/Q-type channels are not a major source of Ca^2+^ for BK activation at either time of day.

**Figure 5.**
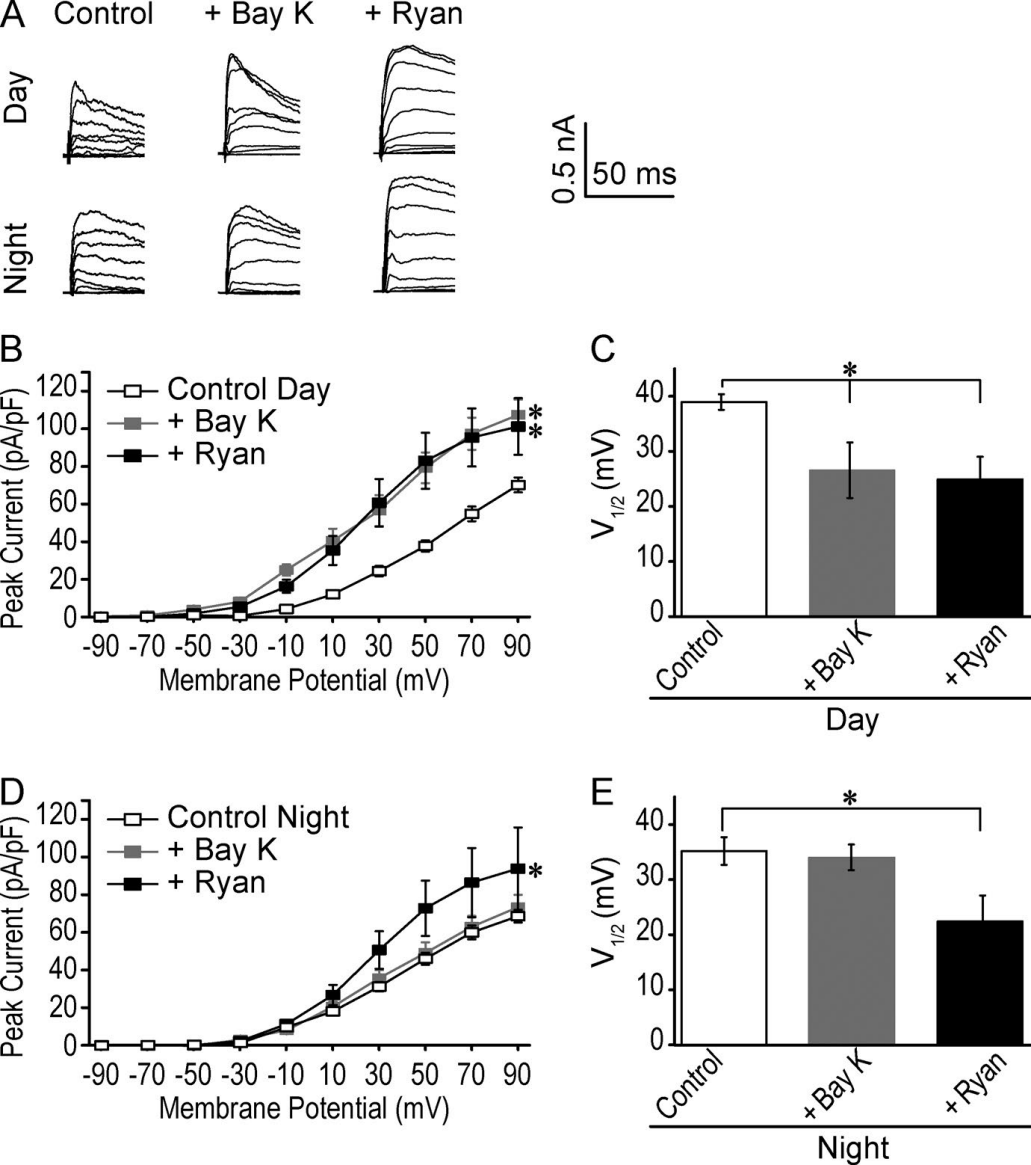
**Effect of agonist activation of Ca^2+^ channels on BK currents. (A)** Representative BK currents recorded in control, 5 µM BayK, and 100 nM Ryan. Voltage protocol same as in Fig. 1. **(B)** Peak current density versus voltage from daytime WT SCN neurons. BayK and Ryan each produced an increase BK current magnitude. *, One-way ANOVA with Bonferroni post hoc test at 90 mV: control (*n* = 24) versus BayK, P = 0.002 (*n* = 8) and Ryan, P = 0.017 (*n* = 7). At 30 mV, BayK and Ryan both significantly increased the BK current (P = 0.001 and 0.001, respectively). **(C)** V_1/2_ values from I/I_max_ relationships. BayK and Ryan produce larger currents via a shift in the voltage dependence of activation to more hyperpolarized membrane potentials. *, One-way ANOVA with Bonferroni post hoc test: control versus BayK, P = 10^−3^ and Ryan, P = 10^−3^. **(D)** Nighttime peak current density versus voltage. Only Ryan produced larger BK currents. *, One-way ANOVA with Bonferroni post hoc test at 90 mV: control versus BayK, P = 0.99 (*n* = 17) and Ryan, P = 0.05 (*n* = 8). At 30 mV, only Ryan significantly increased the BK current (P = 0.03). **(E)** V_1/2_ values, with only Ryan producing larger BK current via a shift to more hyperpolarized membrane potentials. *, One-way ANOVA with Bonferroni post hoc test: Control versus BayK, P = 0.99 and Ryan, P = 0.03. All data are mean ± SE.

Because these data demonstrate that the predominant Ca^2+^ source for BK channel activation in SCN neurons changes with the circadian cycle, from primarily requiring LTCCs during the day, to primarily requiring RyR-mediated Ca^2+^ release at night, it opens the question of whether BK channels may dynamically couple and uncouple from their Ca^2+^ sources over the circadian cycle. To determine whether BK channels become functionally insensitive to LTCC activation at night, or RyR-mediated intracellular Ca^2+^ release during the day, we used agonists to increase the Ca^2+^ from each source during the day and at night. First, 5 µM BayK, the dihydropyridine LTCC agonist (Marcantoni et al., 2010), and 100 nM Ryan, which opens RyRs at nanomolar concentrations (Aguilar-Roblero et al., 2007), were applied during the day. Consistent with the dramatic daytime effect of inhibiting LTCCs (Fig. 3), application of BayK increased the peak daytime BK current by 55% (Fig. 5, A and B) and was accompanied by a shift in the V_1/2_ toward more hyperpolarized potentials (Fig. 5 C). Ryan also increased daytime BK current by 46% (Fig. 5, A–C), despite the reduced impact of Dan on BK current amplitude during the day (Fig. 3). Conversely at night, when inhibition of Ca^2+^ release from intracellular stores, but not L-type Ca^2+^ influx, had the largest effect on BK current activation, only Ryan produced an enhancement of BK current magnitude (33%) and reduced the V_1/2_ values (Fig. 5, D and E). BayK had no effect on nighttime BK current amplitude or V_1/2_ (Fig. 5, D and E). The finding that enhancement of LTCC activity can increase BK current only during the day, but enhancement of RyR-mediated Ca^2+^ channel activity increases BK current at both times of day, supports a model where BK channels become functionally uncoupled from LTCCs at night. In contrast, the functional coupling may not be as strictly regulated between day and night for BK and RyR channels.

### LTCC-mediated BK current activation facilitates BK current inactivation

**Figure 6.**
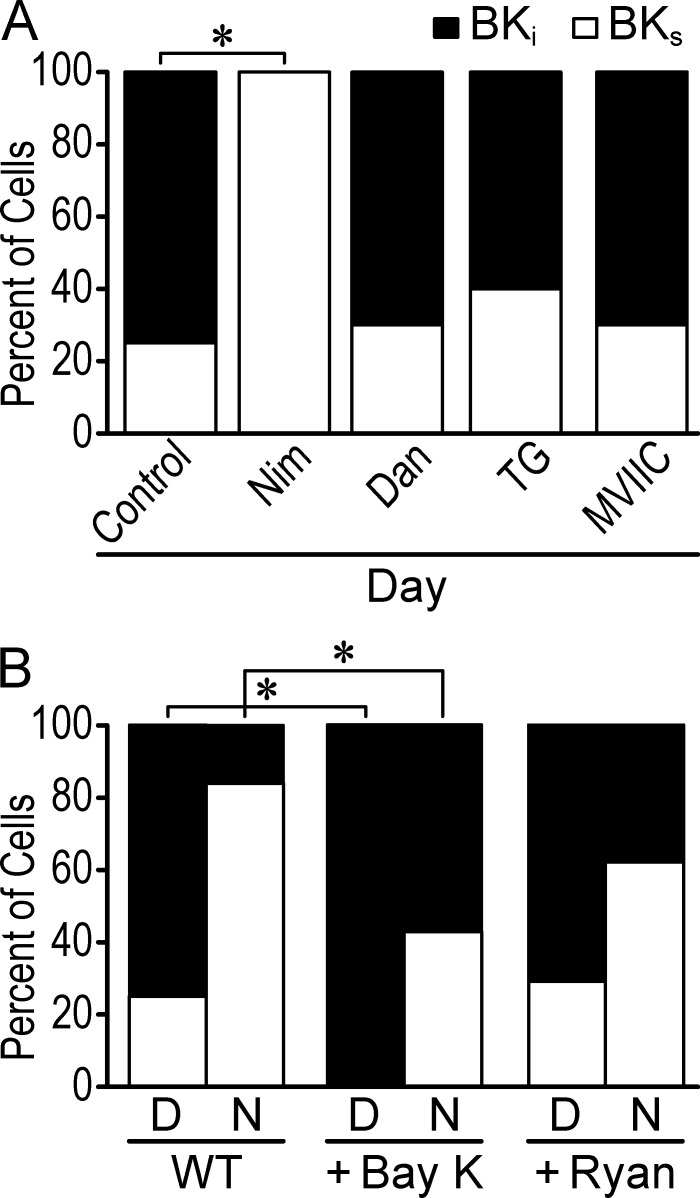
**Effect of inhibition and activation of Ca^2+^ channels on BK current inactivation.** BK currents from daytime WT SCN neurons were categorized as BK_i_ or BK_s_ as described previously (Whitt et al., 2016). **(A)** Percentage of cells with inactivating BK currents in control (*n* = 24), Nim (*n* = 14), Dan (*n* = 17), TG (*n* = 10), and MVIIC (*n* = 10). Nim, but not the other inhibitors, abolished BK_i_ currents during the day. *, P < 0.05, Fisher’s exact test: control versus Nim, P = 10^−4^. **(B)** Percentage of cells with BK_i_ and BK_s_ currents from day and night WT SCN neurons in BayK and Ryan. BayK increased the number of BK_i_ neurons compared with control during day and night, but Ryan had no effect. *, P = 0.04 (day) and 0.039 (night), control versus BayK, Fisher's exact test. Control (day, *n* = 24; night, *n* = 25), BayK (day, *n* = 8; night, *n* = 17), and Ryan (day, *n* = 7; night, *n* = 8).

The daytime dependence of BK current activation on LTCCs raised the question of whether BK channel inactivation, which has a higher prevalence during the day in SCN neurons (Whitt et al., 2016), was regulated by specific Ca^2+^ sources. If LTCC coupling is involved in driving inactivation of BK channels during the day, then Nim and BayK would affect the number of BK_i_ currents, in addition to the overall current magnitude. Consistent with this, in the neurons that retain their BK current in Nim, the proportion of neurons with inactivating macroscopic BK currents changes from 75% in control to zero (Fig. 6 A). This suggests that LTCC activation could be required for BK inactivation. In contrast, Dan, TG, and MVIIC have no effect on the proportion of inactivating BK currents in daytime SCN (Fig. 6 A). These data support a model where BK-LTCC coupling is required to produce the essential channel gating property, daytime inactivation, that underlies BK’s diurnal influence on membrane excitability.

Further supporting the requirement for LTCCs for BK channel inactivation, increasing LTCC activity with BayK was sufficient to convert all daytime BK currents to inactivating (Fig. 6 B). However, at night, analyzing inactivation properties also revealed an increase in the number of neurons with inactivating BK currents, from 16% in control to 48% in BayK (Fig. 6 B). This result suggests that some of the BK channels may still be located in close enough proximity to respond to the extra Ca^2+^ influx through LTCCs, and it contrasts with the results with Nim, suggesting that BK channels might uncouple from LTCCs at night (Fig. 4). With Ryan application, the proportion of BK_i_ currents did not change significantly, either during the day or at night (Fig. 6 B), despite the observed increase in BK current (Fig. 5). Taken altogether, the inhibitor and agonist results suggest a more elaborate model for coupling, where BK activation is regulated by additional factors beyond the binary existence of only BK-LTCC complexes during the day and only BK-RyR complexes at night. However, although the data support that BK activation by LTCCs is linked to BK channel inactivation, it is tempting to speculate that BK activation by RyR-mediated Ca^2+^ results in sustained, noninactivating BK currents.

**Figure 7.**
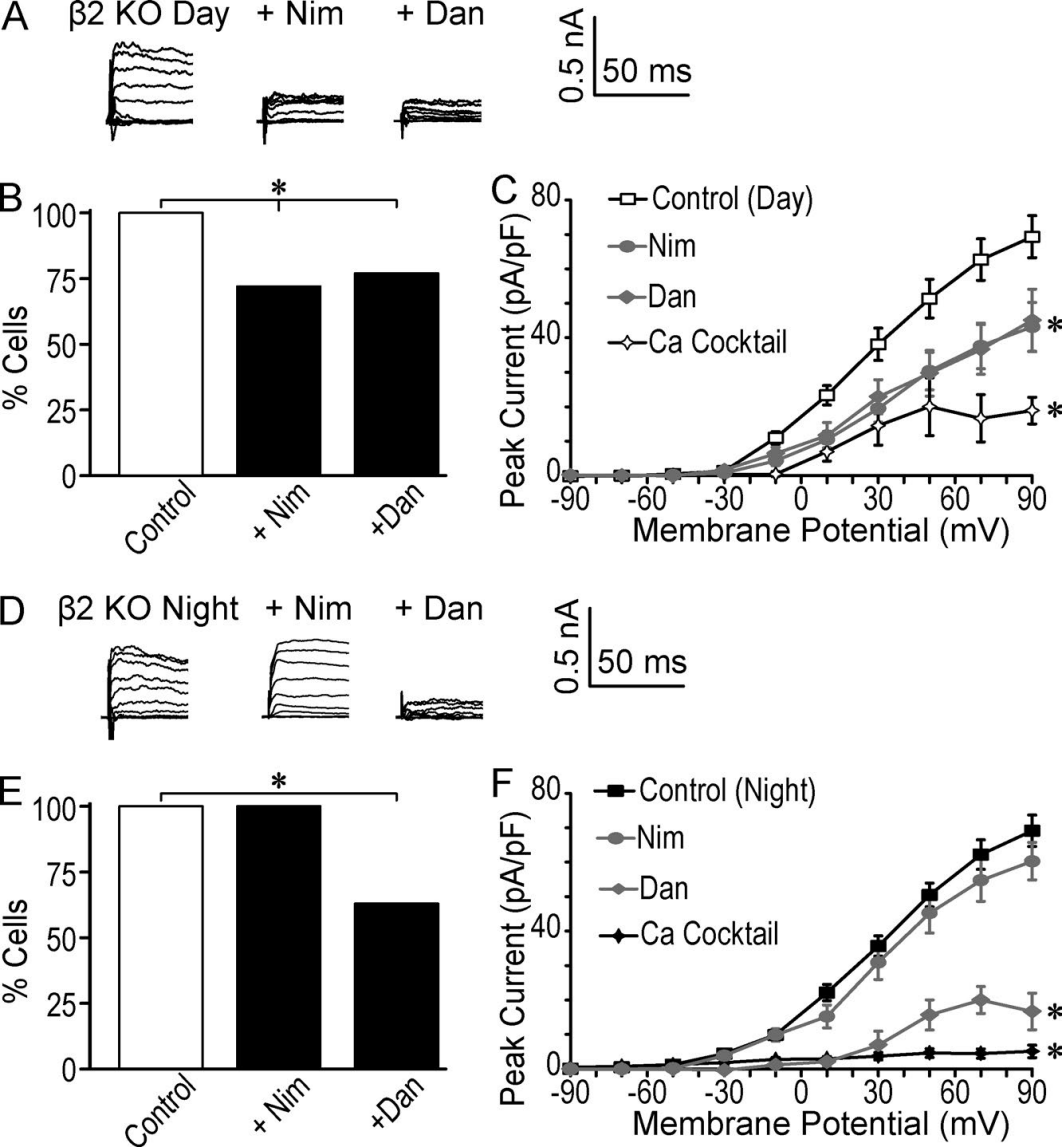
**Effect of inhibition of Ca^2+^ channels on BK currents from β2 KO SCN neurons. (A–C)** BK currents recorded from β2 KO SCN neurons during the day. (A) Representative BK current traces in control (*n* = 22), 10 µM Nim (*n* = 18), and 10 µM Dan (*n* = 13). (B) The percentage of cells with a detectable BK current was reduced with Nim and Dan. *, Fisher's exact test (control vs. Nim, P = 0.01 and Dan, P = 0.04). (C) Peak daytime BK current density versus voltage from only those cells with a BK current in β2 KO control (*n* = 22), Nim (*n* = 13), Dan (*n* = 10), or a cocktail of 10 µM Nim, 10 µM Dan, and 3 µM MVIIC (*n* = 4). In β2 KO neurons, both Nim and Dan decreased BK current magnitude compared with control. Unlike with WT neurons (Fig. 3 D), the Ca^2+^ cocktail did not completely inhibit all BK current. *, One-way ANOVA with Bonferroni post hoc test at 90 mV: control versus Nim, P = 10^−3^; Dan, P = 10^−3^; and Ca^2+^ cocktail, P = 10^−4^). At 30 mV, only Nim significantly decreased the BK current (P = 0.03). **(D–F)** BK currents recorded from β2 KO SCN neurons during the night. (D) Representative BK current traces. (E) The percentage of cells with a detectable BK current was decreased with Dan at night. *, Fisher's exact test (control vs. Dan, P = 10^−4^). β2 KO Control, *n* = 17; Dan, *n* = 8; Nim, *n* = 12; and Ca^2+^ cocktail, *n* = 5. (F) Peak daytime BK current density versus voltage from only those cells with a BK current in control (*n* = 17), Nim (*n* = 12), Dan (*n* = 5), or cocktail (*n* = 5). Dan, but not Nim, produced a large decrement in BK current at night, similar to WT neurons (Fig. 4 D). *, One-way ANOVA with Bonferroni post hoc test at 90 mV: control vs. Dan, P = 10^−4^ or Ca^2+^ cocktail, P = 10^−4^. At 30 mV, Dan and the Ca^2+^ cocktail, but not Nim, significantly decreased the BK current (P = 0.001 and 0.002, respectively). All data are mean ± SE.

The finding that increasing LTCC activity can alter BK inactivation also raises the possibility that the β2 subunit, which confers inactivation of SCN BK currents (Whitt et al., 2016), plays a role in coupling BK channels to specific Ca^2+^ sources. The β2/α expression ratio changes from higher during the day to lower at night, which correlates with the prevalence of inactivating currents in SCN (Whitt et al., 2016). To test whether β2 is required for BK activation by LTCCs, we recorded BK currents under Nim inhibition from β2 KO (*KCNMB2^−/−^*) SCNs (Martinez-Espinosa et al., 2014; Whitt et al., 2016). During the day, Nim both decreased the number of neurons with a detectable BK current and reduced the BK current in the remaining neurons (Fig. 7, A–C). The sensitivity of the BK current in β2 KO neurons to Nim suggests that the β2 subunit is not strictly required for BK channel activation by LTCCs. However, compared with WT (Fig. 3, A and B), the relative inhibition with Nim was changed in β2 KO neurons (Fig. 7, A–C). Nim decreased BK current by about half in β2 KO neurons (Fig. 7 C), which was less than the reduction in WT (Fig. 3 B). Similarly, the number of neurons relying solely on LTCC current for BK activation was 28% in β2 KO neurons (Fig. 7 B), compared with 50% for WT (Fig. 3 C). Concurrently, during the day the relative reduction in BK current with Dan increased to 50% β2 KO neurons (Fig. 7 C), from 22% in WT (Fig. 3 B). Notably in β2 KO neurons, application of the combination of inhibitors (Nim, Dan, and MVIIC) significantly attenuated but did not completely block the activation of daytime BK currents (Fig. 7 C) as it did in WT (Fig. 1 B). The change toward a more equivalent contribution of LTCC and RyR-mediated Ca^2+^ to BK activation during the day in β2 KO neurons compared with WT (Fig. S2) suggests that, although the β2 subunit is not globally required for activation by LTCCs during the day, it may modify coupling by either influencing the physical association between BK and LTCC or RyR channels or the functional response of BK channels to intracellular Ca^2+^ influx.

Recording at night, when most WT BK currents are noninactivating (Fig. 6 B; Whitt et al., 2016), there was little reduction in the overall BK current magnitude after Nim application in β2 KO neurons (Fig. 7, D–F). This corroborates results obtained from WT neurons (Fig. 4). However, using Dan to inhibit RyRs resulted in a larger decrease in β2 KO BK current compared with WT at night, with about the same number of cells where the BK current was solely dependent on RyR-mediated Ca^2+^ (Fig. 7, D–F). These nighttime differences between β2 KO and WT pharmacological sensitivity (Fig. S2) also keep open the possibility that the β2 subunit modifies the BK-Ca^2+^ channel interaction in some way.

### LTCC and RyR regulation of spontaneous action potential firing frequency

**Figure 8.**
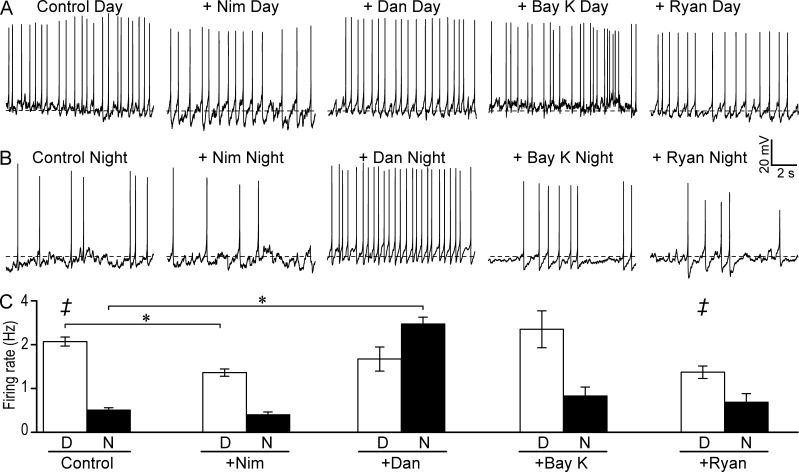
**Effect of inhibition and activation of Ca^2+^ channels on firing rates in SCN neurons. (A and B)** Spontaneous action potential activity from representative daytime (A) and nighttime (B) WT SCN neurons in 10 µM Nim, 10 µM Dan, 5 µM BayK, or 100 nM Ryan. Dotted line denotes −50 mV. **(C)** During the day, both Nim (*n* = 8) and Ryan (*n* = 7) decreased the firing rate compared with the control (*n* = 8). Dan and BayK had no effect on daytime firing. However, at night, Dan increased firing (*n* = 7) compared with the control (*n* = 13), whereas Nim (*n* = 8), BayK (*n* = 7), and Ryan (*n* = 8) had no effect. *, One-way ANOVA with Bonferroni post hoc: control versus Nim (day), P = 0.02; Ryan (day), ‡, P = 0.04; or control versus Dan (night), P = 10^−3^. All data are mean ± SE.

If BK-LTCC and BK-RyR coupling are important contributors to the circadian differences in BK current properties, then inhibition of these Ca^2+^ sources should affect neuronal firing rates at opposing times of the cycle. During the day, Nim decreased firing on average compared with control (Fig. 8, A and C), consistent with previous studies (Pennartz et al., 2002; Cloues and Sather, 2003; Jackson et al., 2004). First, if this effect was solely to the result of inhibiting the inward LTCC current, the decreased firing in Nim should be accompanied by hyperpolarization of the resting membrane potential (Jackson et al., 2004), which was not observed (Fig. S1 A). This suggests that Nim’s effect is not solely through the loss of LTCC current. A second possibility is that this change in firing is a consequence of altering the BK current. In this study, Nim caused a reduction in the number of cells with a detectable BK current, and both a reduction in the BK current and loss of inactivation in the remaining cells. Although the outright loss of BK current might be expected to increase firing, this was not observed. On the other hand, the loss of inactivation of the BK current would be expected to decrease firing, as in the case of the β2 KO SCN neurons, which exhibit decreased daytime firing without inactivation of BK currents (Whitt et al., 2016).

Consequently, if the decrease in firing with Nim was enacted through BK channels, then it should be recovered in the presence of the BK channel inhibitor, Pax. Consistent with this idea, coapplication of Pax partially mitigated the decrease in firing in Nim (control: 2.0 ± 0.14 Hz, *n* = 8; Nim alone: 1.3 ± 0.08 Hz, *n* = 8; Nim + Pax: 1.7 ± 0.20 Hz, *n* = 6; one-way ANOVA, with Bonferroni post hoc: control vs. Nim, P = 0.02). Thus, despite the mixed consequences of LTCC activation that may converge on action potential activity, at least half of Nim’s effect on daytime firing can be attributed to BK channels. Assuming each of these cells had a BK_s_ current in the presence of Nim (Fig. 6 A), the increase in firing with Pax is consistent with BK current inhibition. Furthermore, the decrease in input resistance observed with Nim was recovered by applying Pax (Fig. S1 B). These results suggest that during the day, BK channels could be a secondary effector of LTCC regulation of excitability that is necessary for the normal daytime increase in firing in SCN.

BayK, which both increases peak BK current levels and increases inactivation during the day, has little effect on daytime firing (Fig. 8 C). If it is assumed all cells recorded with BayK were BK_i_ during the day (Fig. 6 B), then inhibition of BK current would not be expected to alter the firing rate. This prediction is consistent with addition of Pax alone, which does not alter firing (control: 2.0 ± 0.14 Hz, *n* = 8 vs. Pax: 1.9 ± 0.20 Hz, *n* = 5; Whitt et al., 2016).

At night, both the decrease in the pharmacological sensitivity of the BK current to LTCC manipulation (Figs. 4 and 5) and the overall decrease in LTCC current (Fig. 2) predict a reduced effect of Nim on SCN firing. Accordingly, no major effect of Nim or BayK on the firing rate at night was observed (Fig. 8, B and C). This lack of change in nighttime firing rate after Nim application was also observed in rat SCN (Pennartz et al., 2002). Concomitant with this lack of effect on firing, Nim did not affect nighttime resting membrane potential or input resistance (Fig. S1, C and D). Thus, the daytime-restricted effect of Nim on firing rate mirrors the daytime-biased role for LTCCs in BK current activation.

Correlated with the shift in BK’s dependence on intracellular Ca^2+^ sources at night (Fig. 4), RyR inhibition would be predicted to alter action potential activity to a greater extent at night. Consistent with this, Dan produced a large increase in the firing rate when applied at night but not during the day (Fig. 8, B and C). In fact, the nighttime firing increase in Dan was elevated to frequencies comparable with those found in the day (Fig. 8 C), an effect consistent with loss of the BK current altogether in the BK KO mice (Meredith et al., 2006). The increase in firing was accompanied by a large depolarization of nighttime membrane potential and increase in input resistance, which were comparable to the values obtained with Pax to inhibit BK channels (Fig. S1, C and D). These results suggest that BK activation by RyRs is critical to suppressing excitability in nighttime SCN neurons.

To more fully explore the relevance of each Ca^2+^ source on excitability, we further assessed changes in firing rate with the agonists BayK and Ryan at the opposite time of day from their predominant effect on BK current levels. Application of BayK had left open the question of whether some BK-LTCC channel complexes remain functionally coupled at night, or whether some BK-RyR remain functionally coupled during the day (Fig. 5). First, with application of BayK at night, when Nim has little effect on BK current levels and firing, there is little effect on firing frequency (Fig. 8, B and C). Thus, despite the increase in the number of neurons with inactivating BK currents in BayK at night (Fig. 6 B), suggesting BK-LTCC complexes may persist at the opposite time of day, the firing rate was not appreciably altered. We conclude from this data that if BK channels do persist in complexes with LTCCs at night, they do not contribute appreciably to the regulation of action potential firing rate.

We next tested whether increasing Ca^2+^ release from stores with Ryan could reduce firing during the day, as predicted for activation of additional BK current (Fig. 5). We found Ryan could reduce daytime firing (Fig. 8, A and C). Thus, in contrast to a model of functional uncoupling for BK-LTCC at night, these data would suggest that during the day, there are BK channels involved in setting action potential frequency that remain close enough to RyRs to be activated by the additional Ca^2+^ release from stores. This is consistent with the effects for both inhibition and activation of RyRs on BK current activation (Figs. 3, 4, and 5). Therefore, there may be a role for RyR-mediated Ca^2+^ release from stores in modulating the firing rate at both times of the cycle under some conditions, and the BK channel is implicated as a candidate mediator for this effect.

## Discussion

### Multiple Ca^2+^ sources contribute to BK current activation in a time-dependent manner

In this study, we identified the Ca^2+^ sources responsible for BK current activation in SCN neurons and found evidence that these Ca^2+^ sources contribute to the day-versus-night difference in BK current properties and action potential firing. We found the majority of BK current activation is dependent on LTCCs during the day, with evidence for lesser functional coupling at night. RyR- and N/P/Q-type channels make a smaller contribution to the daytime activation of BK current. At night, RyRs account for the greatest effect on BK current activation. This central conclusion of decreased LTCC involvement, and predominant RyR contribution, to the nighttime activation of BK current was also substantiated on the β2 KO background.

The results rule out a model where circadian regulation of a single Ca^2+^ source is required for the day-versus-night activation of BK current. These findings also resolve a long-standing puzzle concerning the anti-phase relationship between the daytime increase in cytosolic Ca^2+^_i_ versus the nighttime increase in BK current magnitude. The source of these daily cytosolic oscillations has not been fully established, but estimates for the resulting changes in Ca^2+^_i_ vary between 60 and 135 nM during the day and night (Colwell, 2000; Enoki et al., 2012). These values are too low to predict they would support appreciable activation of BK current, which in most cells relies on a local influx of Ca^2+^ to achieve the micromolar levels capable of activating BK channels (Fakler and Adelman, 2008). Instead, multiple Ca^2+^ channel subtypes contribute the intracellular Ca^2+^ necessary for BK activation, in a time-of-day–dependent manner (Fig. S3).

Although our data cannot distinguish the resolution of individual channel interactions within the cell, the results suggest that there are broadly at least two types of SCN neurons—those where BK channels are activated exclusively by a single Ca^2+^ source, which lose all BK current when that source is inhibited, and those where BK channels are activated by multiple sources, which display reduced BK current in the presence of individual inhibitors. This model based on multifarious coupling could account for some of the heterogeneity observed in this study in the peak BK current values between neurons recorded at the same time point and may also factor into the difference in the sensitivity of the BK current to the nonselective VGCC blocker cadmium observed in a prior study (Pitts et al., 2006). In addition, the data in this study do not definitively ascertain whether BK channels and LTCCs fully uncouple at night. Although both the agonist and antagonist effects on BK current levels and firing activity support this conclusion, the increase in the number of inactivating neurons after BayK treatment at night unexpectedly suggests that BK channels are still sensitive in one respect to an increased LTCC current. However, the mechanism for how BK channels become functionally insensitive to LTCC current at night under normal conditions remains to be determined.

### Role of Ca^2+^ in circadian regulation of BK current properties

The factors that specify the changeover in BK activation from primarily LTCC during the day to primarily RyR-mediated Ca^2+^ release at night are not fully clear. One possibility is that because of the decrease in LTCC current at night (Fig. 2; Pennartz et al., 2002), an alternative Ca^2+^ source is necessary to maintain the higher steady-state nighttime levels of BK current. A reduction in LTCC current could stem from removal of these channels from the plasma membrane, reducing the number of functionally coupled BK channels (Fig. S3). However, the molecular mechanisms behind the nighttime reductions in VGCC and LTCC currents have not yet been established (Pennartz et al., 2002).

A second possibility is that each Ca^2+^ source is required in a time-of-day–specific manner to regulate additional aspects of SCN neuronal physiology, mechanisms which could have been coopted by BK channels to receive the necessary Ca^2+^ for activation at each time of day. However, whereas inhibition of LTCCs clearly has a daytime-restricted effect on firing (Fig. 8; Pennartz et al., 2002), during the night at least one subtype of LTCC has been shown to continue to mediate some aspects of SCN physiology. Cav1.2 expression was reported to peak during the late night and be involved in late-night phase advances in circadian behavior (Kim et al., 2005; Schmutz et al., 2014). Another LTCC subtype, Cav1.3, is also expressed in SCN and has been shown to undergo RNA editing (Huang et al., 2012). Moreover, disruption of RNA adenosine deaminase ADAR2 activity alters circadian firing patterns (Huang et al., 2012), but the time-of-day dependence is still an open question.

A comprehensive understanding of the release of Ca^2+^ from intracellular stores between day and night in SCN is also lacking, confounding development of a detailed model for how this source interacts with BK channels over the circadian cycle. RyR expression is clock-linked (Pfeffer et al., 2009), and Ryan binding is rhythmic (Díaz-Muñoz et al., 1999). However, this rhythm has been reported to be daytime phased and suggested to contribute to the cytosolic Ca^2+^_i_ oscillation observed in Ca^2+^ imaging studies (Ikeda et al., 2003). On the other hand, RyR Ca^2+^ has also been reported to be important for light-induced phase delays at night (Ding et al., 1998; Pfeffer et al., 2009). Thus, like LTCCs, it is not yet established whether RyR-mediated Ca^2+^ contributes to SCN signaling in a time-delimited manner. Furthermore, during either the day or night, there are currently no studies addressing RyR-dependent local Ca^2+^ signaling domains in SCN neurons.

A third possibility for the factors that specify the changeover in BK activation is that specific Ca^2+^ sources are required to specify day-versus-night properties of the BK current. The central circadian difference in BK current properties is inactivation (Whitt et al., 2016). In contrast to the BK activation from the initial (peak) current investigated in this study, in previous work we established that as a result of inactivation, steady-state BK current has a more pronounced circadian difference. Steady-state BK current levels are significantly lower during the day and higher at night (Meredith et al., 2006; Pitts et al., 2006; Farajnia et al., 2015; Whitt et al., 2016). This daytime BK channel inactivation is required to allow depolarization of the membrane before threshold, which facilitates the daytime increase in the SCN firing rate (Whitt et al., 2016). The results obtained in this study suggest that the importance of LTCCs in daytime BK current activation is to facilitate this BK current inactivation. Yet despite this link, it is clear that the β2 subunit is not absolutely required to partner BK channels with this Ca^2+^ source (Fig. 7). Consistent with this, β subunits are not required to reconstitute interactions between BK and VGCCs in heterologous expression systems (Berkefeld et al., 2006; Berkefeld and Fakler, 2008; Vivas et al., 2017).

Following this idea into the night, when BK inactivation is largely reduced (Whitt et al., 2016), a more sustained and less voltage-dependent source of intracellular Ca^2^ may be necessary to activate BK channels. Nighttime membrane potentials are more negative, yet BK current is activated during the long interspike intervals that occur at nighttime firing frequencies (Whitt et al., 2016). We speculate that RyR-mediated Ca^2+^ release may facilitate activation of this interspike current at subthreshold membrane potentials. Although the activation of BK channels by RyR or IP3-mediated Ca^2+^ release from intracellular stores has been well studied in smooth muscle (Nelson et al., 1995; Jaggar et al., 1998; Brenner et al., 2000b; ZhuGe et al., 2000, 2002; Pérez et al., 2001), it has also been more recently appreciated in neurons where RyR-mediated Ca^2+^ contributes to BK current activation on rapid time scales during the action potential (Chavis et al., 1998; Parsons et al., 2002; Beurg et al., 2005; Wang et al., 2016; Irie and Trussell, 2017). This raises the question of what functions as the sensor for RyR Ca^2+^ release. The data in this study would suggest L- or P/Q-type Ca^2+^ channels are not involved in RyR-mediated Ca^2+^-induced Ca^2+^ release in SCN, as has been proposed for other neurons (Chavis et al., 1996; Mouton et al., 2001; Verkhratsky, 2005; Irie and Trussell, 2017).

Although overall the individual roles and interaction of these two Ca^2+^ sources for SCN physiology or excitability is not yet well understood, the dependence of BK activation during the action potential on multiple membrane and intracellular Ca^2+^ sources has been observed in other cells (Prakriya et al., 1996; Chavis et al., 1998; Prakriya and Lingle, 1999; Akita and Kuba, 2000; Smith et al., 2002; Beurg et al., 2005; Goldberg and Wilson, 2005; Loane et al., 2007; Marcantoni et al., 2010; Wang et al., 2016; Irie and Trussell, 2017). Still, it is not currently known how multifactorial coupling is specified in the context of any native cell, despite the direct interactions demonstrated between BK channels and Ca^2+^ channels (Grunnet and Kaufmann, 2004; Berkefeld et al., 2006; Loane et al., 2007; Berkefeld and Fakler, 2008; Vivas et al., 2017). The presumably more static coupling scenario between BK and its Ca^2+^ sources in other types of cells stands in contrast to the SCN, which undergoes periodic alterations in BK expression levels (Meredith et al., 2006). Moreover, the synthesis of up to 10% of transcripts is circadianly regulated in SCN (Panda et al., 2002), providing a basis for speculating how BK channels could dynamically convert their coupling between different Ca^2+^ sources between day and night (Fig. S3).

### BK channels as membrane effectors of Ca^2+^ in SCN neuronal excitability

The role of Ca^2+^ in SCN intracellular signaling is currently an open question. Ca^2+^_i_ is subject to circadian regulation in SCN neurons (Colwell, 2000; Pennartz et al., 2002; Ikeda et al., 2003; Enoki et al., 2012, 2017a,b; Hong et al., 2012; Brancaccio et al., 2013), but its interaction with membrane events that drive the daily oscillation in action potential activity has not been clear (Ikeda et al., 2003; Enoki et al., 2012, 2017a). Action potentials are not required to generate the cytosolic Ca^2+^_i_ rhythms (Ikeda et al., 2003; Enoki et al., 2012, 2017a; Hong et al., 2012), suggesting regulation of membrane excitability is unlinked from these rhythms and may depend more on local Ca^2+^ signaling. BK channels are positioned to bridge this gap by virtue of their Ca^2+^-dependent activation and central role in the circadian oscillation in SCN firing (Meredith et al., 2006; Colwell, 2011; Montgomery et al., 2013; Whitt et al., 2016).

The results here identify that LTCCs, and the more novel finding that neuronal RyRs, may both contribute to SCN excitability through activation of BK channels. Supporting this hypothesis, the direction and time windows of Dan’s effect on firing matches the basic predictions for being mediated by BK channels (Fig. 8). However, both Nim and Dan have been previously reported to produce bidirectional effects on membrane properties and firing rate on a cell-by-cell basis (Jackson et al., 2004; Aguilar-Roblero et al., 2007, 2016). Ca^2+^ influx through either of these sources could alter cellular excitability through multiple mechanisms. Our results suggest these mixed effects could be partially accounted for by considering whether the cells coexpress BK channels and whether those channels inactivate. For example, although part of the decrease in firing with Nim was recoverable by inhibiting BK channels, LTCCs likely also contribute to higher daytime firing by directly regulating membrane depolarization (Jackson et al., 2004). Because BK current has been shown to be activated at multiple phases of the action potential in SCN neurons (during the interspike interval, repolarization, and afterhyperpolarization; Jackson et al., 2004; Montgomery et al., 2013; Whitt et al., 2016), sorting out the relative basis for how LTCC versus RyR Ca^2+^ sources contribute to action potential firing rate through BK channels will require future studies.

Taken altogether, despite the changes in the firing rate with LTCC and RyR inhibition reported here and in previous studies, independently, neither LTCC nor RyR inhibition fully eliminates the day-versus-night difference in firing or SCN rhythmicity (Pennartz et al., 2002; Ikeda et al., 2003; Kononenko et al., 2008; Ikeda and Ikeda, 2014; Enoki et al., 2017a) like the loss of BK channel activity does (Meredith et al., 2006; Kent and Meredith, 2008). This suggests the dual Ca^2+^-dependent activation pathways converging on BK are strategically positioned to preserve channel activity over the circadian cycle.

## Supplementary Material

Supplemental Materials (PDF)
