# Supplementary material for: Differential contribution of Ca2+ sources to day and night BK current activation in the circadian clock
Source: J Gen Physiol. 2018 Feb 5;150(2):259–75. doi: 10.1085/jgp.201711945 (PMC5806683; doi:10.1085/jgp.201711945)
Supplement: Supplemental Materials (PDF) [file JGP_201711945_sm.pdf]

SUPPLEMENTAL MATERIAL

Whitt et al., https://doi.org/10.1085/jgp.201711945

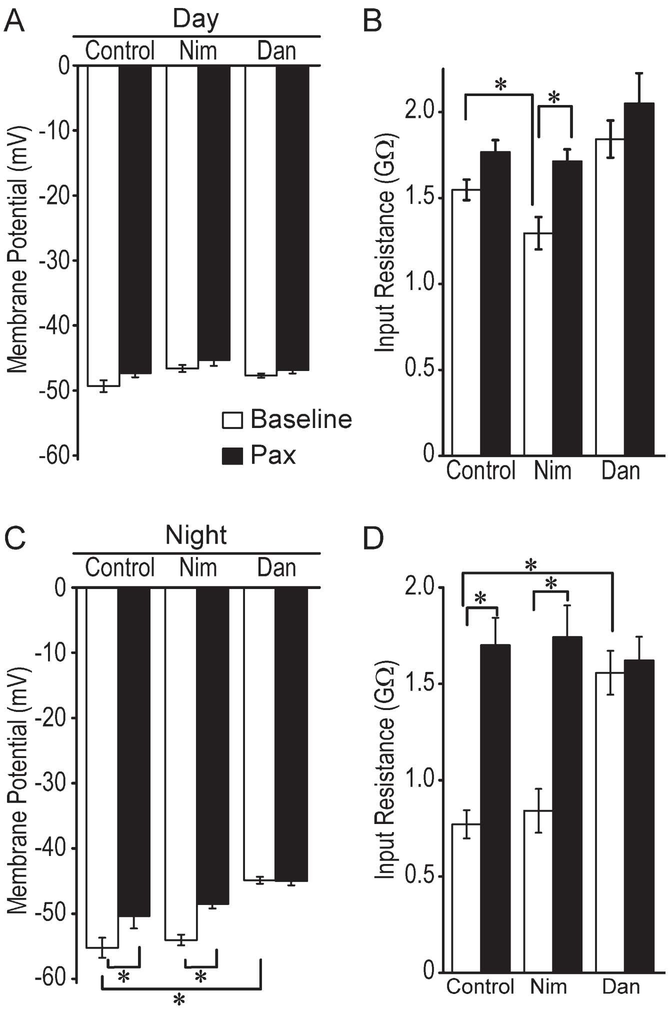

Figure S1. **Effect of inhibition of Ca^{2+} channels on passive membrane properties during the day and night.** Because BK current contributes to the day-versus-night difference in resting membrane potential and input resistance (Whitt et al., 2016), inhibition of a Ca^{2+} source relevant for BK activation should also affect these parameters. **(A–D)** To test this prediction, resting membrane potentials (A, C) and input resistance (B, D) were recorded in 1 µM TTX before and after the addition of 10 µM Pax. Nim was predicted to have its major effects during the day, whereas Dan would have its major effects at night. (A) During the day, Nim and Dan had no significant effect on resting membrane potential, and Pax had no further effect ($P > 0.05$, one-way ANOVA). This effect is consistent with the daytime inactivation of BK channels, which is dependent on LTCCs but not RyRs. (B) During the day, Nim decreased input resistance, which was recovered with addition of Pax. This could be interpreted as a consequence of loss of BK current inactivation with Nim. With Dan, there was a trend toward increased input resistance, potentially indicative of the small role RyRs play in daytime BK current activation. Pax produced no further effect. *, $P < 0.05$, one-way ANOVA with Bonferroni post hoc: control versus Nim, $P = 0.02$; Nim versus Nim + Pax, $P = 10^{-3}$. (A and B) Control: *n* = 20, Nim: *n* = 8, and Dan: *n* = 7. (C) During the night, Dan produced a large depolarization of the resting membrane potential, comparable to the values produced in the presence of BK inhibition by Pax. This suggests that the BK channels mediating the nighttime hyperpolarization of the membrane depend on RyRs for activation. In contrast, Nim had no effect on resting membrane potential compared with control. However, the addition of Pax to both control and Nim leads to depolarization, indicating that BK channels are present, but they appear to be functionally insensitive to LTCC activation. *, $P < 0.05$, one-way ANOVA with Bonferroni post hoc: control versus control + Pax, $P = 0.04$; Nim versus Nim + Pax, $P = 10^{-4}$; control versus Dan, $P = 10^{-4}$; Dan versus Dan + Pax, $P = 0.42$. (D) Consistent with the resting membrane potential results, at night Dan increased input resistance to the same level observed when BK channels were inhibited by Pax. Nim did not produce an effect, despite the presence of BK channels that underlie the increase in input resistance with Pax. *, $P < 0.05$, one-way ANOVA with Bonferroni post hoc: control versus control + Pax, $P = 10^{-4}$; Nim versus Nim + Pax, $P = 10^{-4}$; control versus Dan, $P = 10^{-4}$; Dan versus Dan + Pax, $P = 0.71$. (C and D) Control: *n* = 18, Nim: *n* = 8, Dan: *n* = 7, BayK: *n* = 7, and Ryan: *n* = 7. All values are mean ± SE.

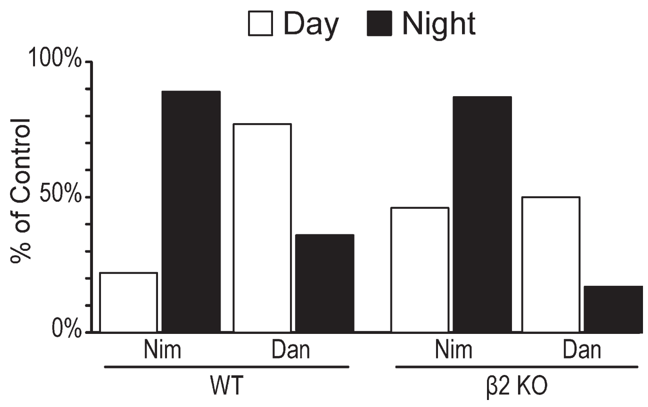

Figure S2. **Comparison of LTCC and RyR Ca^{2+} channel inhibition on the diurnal variation in BK current.** Mean decrease in BK current with Nim and Dan from WT and β2 KO neurons, reported as percent of control (no drug) condition. The means are derived from data in Figs. 3, 4, and 7.

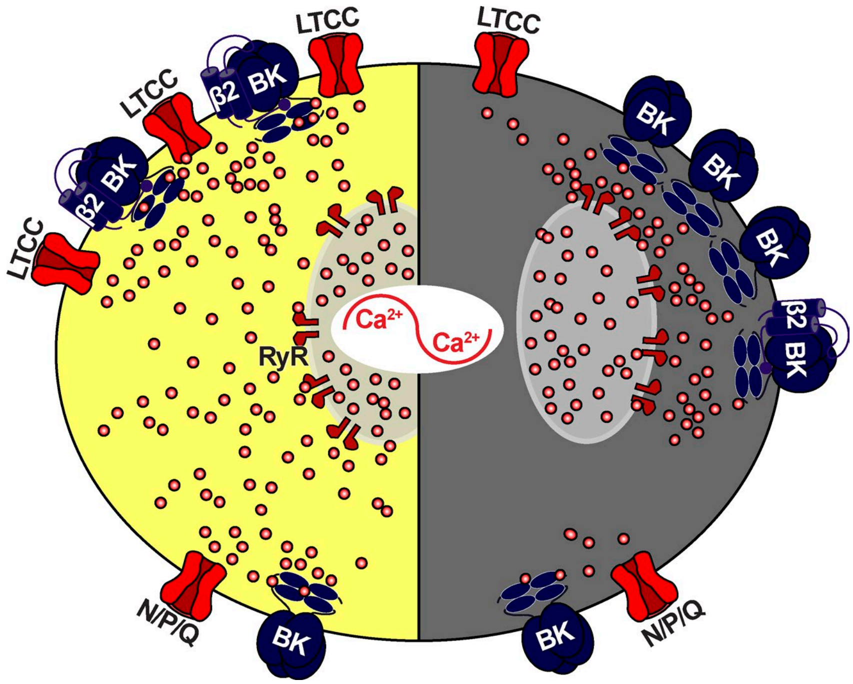

Figure S3. **Hypothesis for BK and Ca^{2+} channel coupling between day and night in SCN neurons.** Intracellular Ca^{2+} is diurnally regulated in SCN neurons, higher during the day than during the night (Colwell, 2000; Ikeda et al., 2003; Enoki et al., 2012; Hong et al., 2012; Brancaccio et al., 2013; Ikeda and Ikeda, 2014). Yet current evidence does not directly implicate Ca^{2+}_i rhythms in the regulation of membrane excitability (Ikeda et al., 2003; Hong et al., 2012), suggesting that Ca^{2+} regulates excitability by acting through more localized signaling domains. The data in this study provide a framework for the dynamic coupling of BK channels in localized signaling domains. These domains are hypothesized to include BK channels colocalized with both plasma membrane and intracellular Ca^{2+} channels, the composition of which would change over the daily cycle. During the day, most daytime BK channels are predicted to contain the inactivating β2 subunit (Whitt et al., 2016) and be predominantly localized close to LTCCs. The data suggests that the β2 subunit is not required for this interaction, but may modify it in some way. Although some cells are predicted to solely rely on LTCCs, in other cells some BK channels would also be colocalized with N/P/Q-type and RyR channels to account for the small, but detectable, BK current reductions produced by their antagonists, and the increase in daytime BK current produced by Ryan. At night, BK-LTCC partnering is hypothesized to be greatly reduced. Based on the decrease in LTCC current at night, a reduction in the number of LTCCs at the membrane could partially account for some of the decreased BK-LTCC coupling at night. The subset of neurons that lose LTCC-dependent activation of BK current at night may be partly the same subpopulation that becomes exclusively RyR-dependent. In addition, when BK channel protein synthesis increases at night (Meredith et al., 2006), these additional channels could be inserted preferentially next to RyRs. Similar to daytime, a small subset of BK channels may remain statically partnered with N/P/Q-type Ca^{2+} channels at night.

REFERENCES

Brancaccio, M., E.S. Maywood, J.E. Chesham, A.S. Loudon, and M.H. Hastings. 2013. A Gq-Ca^{2+} axis controls circuit-level encoding of circadian time in the suprachiasmatic nucleus. *Neuron*. 78:714–728. https://doi.org/10.1016/j.neuron.2013.03.011

Colwell, C.S. 2000. Circadian modulation of calcium levels in cells in the suprachiasmatic nucleus. *Eur. J. Neurosci*. 12:571–576. https://doi.org/10.1046/j.1460-9568.2000.00939.x

Enoki, R., S. Kuroda, D. Ono, M.T. Hasan, T. Ueda, S. Honma, and K. Honma. 2012. Topological specificity and hierarchical network of the circadian calcium rhythm in the suprachiasmatic nucleus. *Proc. Natl. Acad. Sci. USA*. 109:21498–21503. https://doi.org/10.1073/pnas.1214415110

Hong, J.H., B. Jeong, C.H. Min, and K.J. Lee. 2012. Circadian waves of cytosolic calcium concentration and long-range network connections in rat suprachiasmatic nucleus. *Eur. J. Neurosci*. 35:1417–1425. https://doi.org/10.1111/j.1460-9568.2012.08069.x

Ikeda, M., and M. Ikeda. 2014. Bmal1 is an essential regulator for circadian cytosolic Ca^{2+} rhythms in suprachiasmatic nucleus neurons. *J. Neurosci*. 34:12029–12038. https://doi.org/10.1523/JNEUROSCI.5158-13.2014

Ikeda, M., T. Sugiyama, C.S. Wallace, H.S. Gompf, T. Yoshioka, A. Miyawaki, and C.N. Allen. 2003. Circadian dynamics of cytosolic and nuclear Ca^{2+} in single suprachiasmatic nucleus neurons. *Neuron*. 38:253–263. https://doi.org/10.1016/S0896-6273(03)00164-8

Meredith, A.L., S.W. Wiler, B.H. Miller, J.S. Takahashi, A.A. Fodor, N.F. Ruby, and R.W. Aldrich. 2006. BK calcium-activated potassium channels regulate circadian behavioral rhythms and pacemaker output. *Nat. Neurosci*. 9:1041–1049. https://doi.org/10.1038/nn1740

Whitt, J.P., J.R. Montgomery, and A.L. Meredith. 2016. BK channel inactivation gates daytime excitability in the circadian clock. *Nat. Commun*. 7:10837. https://doi.org/10.1038/ncomms10837
